# Participant Perceptions of Facilitators and Barriers to Adherence in a Digital Mental Health Intervention for a Nonclinical Cohort: Content Analysis

**DOI:** 10.2196/25358

**Published:** 2021-04-14

**Authors:** Melanie Elise Renfrew, Darren Peter Morton, Maria Northcote, Jason Kyle Morton, Jason Scott Hinze, Geraldine Przybylko

**Affiliations:** 1 Lifestyle Medicine and Health Research Centre Avondale University College Cooranbong, NSW Australia

**Keywords:** web-based mental health, health promotion, eHealth, adherence, participant perceptions, mobile phone

## Abstract

**Background:**

Digital mental health promotion interventions (MHPIs) present a scalable opportunity to attenuate the risk of mental health distress among nonclinical cohorts. However, adherence is frequently suboptimal, and little is known about participants’ perspectives concerning facilitators and barriers to adherence in community-based settings.

**Objective:**

This study aimed to examine participants’ perceptions of facilitators and barriers to adherence in a web- and mobile app–based MHPI for a nonclinical cohort.

**Methods:**

This qualitative study used inductive, reflexive thematic analysis to explore free-text responses in a postintervention evaluation of a 10-week digital MHPI. The intervention was administered using a web and mobile app from September to December 2018. Participants (N=320) were Australian and New Zealand members of a faith-based organization who self-selected into the study, owned a mobile phone with messaging capability, had an email address and internet access, were fluent in English, provided informed consent, and gave permission for their data to be used for research. The postintervention questionnaire elicited participants’ perceptions of facilitators and barriers to adherence during the intervention period.

**Results:**

Key factors that facilitated adherence were engaging content, time availability and management, ease of accessibility, easy or enjoyable practical challenges, high perceived value, and personal motivation to complete the intervention. The primary perceived barrier to adherence was the participants’ lack of time. Other barriers included completing and recording practical activities, length of video content, technical difficulties, and a combination of personal factors.

**Conclusions:**

Time scarcity was the foremost issue for the nonclinical cohort engaged in this digital MHPI. Program developers should streamline digital interventions to minimize the time investment for participants. This may include condensed content, optimization of intuitive web and app design, simplified recording of activities, and greater participant autonomy in choosing optional features. Nonetheless, participants identified a multiplicity of other interindividual factors that facilitated or inhibited adherence.

## Introduction

### Background

Mental distress and disorders are increasingly prevalent [1], and mental health promotion interventions (MHPIs) present opportunities to enhance the well-being of nonclinical groups, decreasing the risk of mental health distress. The widespread availability of digital technology offers unprecedented accessibility, portability, and cost-effectiveness for implementing such interventions on a wide scale. However, adherence, defined by Kelders et al [2] as “the extent to which individuals should experience the content (of the intervention) to derive maximum benefit from the intervention, as defined or implied by its creators,” is often suboptimal [3], and little is known about participants’ perceptions of facilitators of and barriers to adherence for nonclinical cohorts in community-based settings.

Considerable research has been undertaken by researchers and program developers to facilitate optimum adherence to digital health initiatives. Methods used to promote participant adherence include the adoption of a robust set of persuasive system design principles [2,4,5], provision of rewards and incentives, positive feedback [6], implementation of gamification techniques [7,8], and various forms of human support [9]. However, adherence to digital interventions remains to be problematic [3], and there is a paucity of research elucidating participant perspectives on the factors that impact adherence [10] in community-based, nonclinical groups. Importantly, factors that may hamper adherence in a clinical population (eg, disease and disorder symptoms) [11,12] may be dissimilar to the influences on adherence in healthy nonclinical cohorts.

Several qualitative studies have addressed participant perspectives on perceived barriers to and facilitators of adherence in MHPIs for nonclinical cohorts in workplace settings. A stress management intervention, using mindfulness training, found that participants’ attitudes (eg, motivation, previous interest in mindfulness, and positivity toward change), awareness of treatment effectiveness, curiosity, and alignment with personal preferences for managing stress were facilitators of adherence [13]. Conversely, barriers included insufficient detail or evidence provided as a rationale for the strategy used, length of time required for completing activities, and program intensity [13]. Carolan and de Visser [14] explored employee perspectives on workplace digital mental health interventions and found that lack of time was considered the core barrier to adherence. Notably, anonymity, convenience, and flexibility were deemed to have dual impacts (ie, considered both a facilitator and barrier). Employees described their ideal workplace intervention as one that was short, that was easily accessible, that was available for an indefinite period, updated regularly, and that provided some form of e-coaching support [14]. Research by Blankenhagel et al [15], which focused on the perspectives of various stakeholders in digital stress management programs, found that certain requirements of interventions were needed for nonclinical users who often have substantial work responsibilities. These intervention requirements were prioritizing time efficiency (5- to 15-minute sessions), splitting larger goals into smaller goals, providing flexibility for users to design their own personal notification or reminder schedule, featuring an intuitive and simple web or app design, prioritizing individualization (ie, tailoring), providing high autonomy, fostering some degree of human interaction, facilitating comparability to peers, and offering high mobility [15]. The aforementioned requirements related to a work-based setting for a nonclinical cohort; however, although there may be similarities, variant influences on adherence may be present in a community-based context.

This study sought to broaden the understanding of factors that both facilitate and hinder adherence in a nonclinical cohort participating in a digital MHPI within a community-based situation. This study builds on quantitative data collected during a randomized comparative study that examined the impact of different levels of human support on well-being outcomes [16] and attrition and adherence [17] in a digital MHPI for a nonclinical cohort. Quantitative data revealed improvements in mental well-being irrespective of the level of human support offered, and adherence did not differ significantly between groups. Notably, using the aforementioned definition of adherence by Kelders et al [2], participants were advised to view one video weekly and complete daily and weekly experiential activities over a 10-week period to gain optimal benefit from the intervention. Overall, attrition and adherence were suboptimal [17]. A total of 605 subjects indicated an interest in the intervention by filling out a preliminary web-based enrollment form, with 24.3% (147/605) of these subjects not fully registering by failing to complete the prequestionnaire. Of those who completed the prequestionnaire (n=458), a further 30.1% (138/458) did not complete the postquestionnaire, resulting in a completion rate of 69.9% (320/458). Primary adherence was measured by the number of videos viewed, with 34% (109/320) of the cohort watching less than half of the videos, 18.9% (60/320) viewing 5 to 9 videos, and 47.1% (151/320) viewing all 10 videos. Secondary adherence was measured by the points scored for completing assigned daily and weekly activities. Participants could score a total of 1000 points (ie, 100 points weekly throughout the 10-week intervention) to be considered fully adherent. The mean score for the cohort was 362, indicating low adherence to challenge activities [17].

### Objectives

This study aims to increase knowledge regarding participant adherence in digital MHPIs by revealing participant perceptions about the facilitators and barriers to watching the videos and their completion of daily and weekly experiential activities. The findings will assist researchers and developers to refine and improve the design and implementation of interventions to optimize the adherence of participants in future MHPIs in nonclinical settings.

## Methods

### Overview

This qualitative study stems from the aforementioned study, which reported the quantitative results of the intervention [16,17]. These qualitative responses were recorded as part of the postquestionnaire administered at the conclusion of the intervention. Participants were allocated a 2-week period, immediately after the intervention period, to complete the postquestionnaire on either the provided app or the web platform. Therefore, the following section of this paper is a summary of the methods used in the aforementioned study, with details only provided for those areas related to the qualitative components of this study.

### Setting and Participants

Advertising was used to direct potential participants (Australian and New Zealand members of the Seventh-day Adventist Church) to a website that explained the intervention, outlined the inclusion criteria, and provided an opportunity to self-enroll as a potential participant. To be included in the study, subjects were required to be aged above 18 years, own a mobile phone with SMS text messaging capability, have internet access, be an Australian or New Zealand resident, be fluent in English, provide informed consent, and give permission for their data to be used for research [16]. Participants who completed the MHPI were invited to provide free-text responses to 2 statements within the postquestionnaire.

### Intervention

The intervention program, known as the *Live More Project* or *Lift Project*, introduced participants to aspects of neuroscience and a combination of evidence-based strategies to enhance mental well-being from the disciplines of lifestyle medicine and positive psychology. Designed to be implemented as a weekly session for 10 weeks, the content of the program was shared in a 30-minute audiovisual presentation followed by participation in daily and weekly practical activities to consolidate learning. Video sessions were released weekly with a lock/unlock system so that participants watched the presentations in sequential order. An intervention and activity overview is provided in Multimedia Appendix 1.

Subjects chose to access the program on a web-based learning management system (eLMS) or a mobile app. Along with the audiovisual content, the eLMS or mobile app included a place to complete the pre- and postquestionnaire, a section to log experiential activity and earn points, an opportunity to interact with other participants through a public feed, and gamification strategies (eg, points on a leaderboard or badges) to induce adherence. As an optional extra, previously introduced experiential activities could be repeated and logged in forthcoming weeks to earn extra points. This resulted in an increasingly longer list of activities to complete as the intervention progressed. Screenshots of the web- and mobile app design are shown in Multimedia Appendix 2.

Participants were divided into 3 groups that differed according to the mode of human support allocation. The first group received automated email support only, the second group received automated email support along with text messages (2-3 times weekly), and the third group received weekly videoconference support along with email support [16].

### Data Collection

After completing the intervention, each participant was asked to complete the postquestionnaire within a 2-week period on either the web or mobile app. As part of the postquestionnaire, participants responded to 2 adherence-related statements in an expanding text box with no imposed word limits. These statements were as follows:

- *Statement 1*: thinking about your personal experience as a participant in the Live More Project over the last 10 weeks, if it was *easy* for you to watch the video presentations and complete the daily and weekly challenges, please describe/list all the factors that contributed to this.
- *Statement 2*: thinking about your personal experience as a participant in the Live More Project over the last 10 weeks, if it was *difficult* for you to watch the video presentations and complete the daily and weekly challenges, please describe/list all the factors that contributed to this.

### Data Analysis

An inductive, reflexive thematic analysis (RTA) approach [18-20] was used to extract meaning from the data set. The inductive approach is sometimes described as *bottom-up*, or data-driven, as the themes are generated from within the data [20]. The RTA method suited one researcher (MR) performing the initial coding, followed by 2 additional coders reviewing the progress. MR had multiple roles in the study and functioned as the technical support person; therefore, this approach, in which multiple coders reviewed the data (as recommended by O’Brien et al [21]), reduced the possibility of personal bias.

The coding researcher adopted the 6 phases of thematic analysis by Braun and Clarke [20] as a structured approach and used a spreadsheet (Microsoft Excel) and a qualitative analysis program (NVivo software) to assist in collation, analysis, and coding of the deidentified data. All identifying information was removed before coding, and participant identification numbers were used instead. In phase 1, the researcher read through the data set twice to maximize familiarity with the data. During phase 2, all comments were systematically analyzed by constructing and labeling codes in a descriptive and interpretive manner until all data were coded. In an additional immersion stage, coding was reviewed and code names were edited. In phase 3, the researcher scrutinized the codes for patterns, relationships, and similarities. The codes were clustered together based on broad themes. During phase 4, after repeated scrutiny to ensure that the code names accurately matched the data and that the developing themes represented the participants’ descriptions of their most typical experiences, clusters were formed into broad themes and several subthemes to develop a thematic map, as recommended by Braun and Clarke [18]. To ensure that the themes and subthemes accurately reflected the original data set as a whole, and the coded data, a final revision of the themes and codes was undertaken by the original coding researcher in consultation with 2 other researchers. During this process, the coders analyzed the themes and subthemes in relation to the original intention of the study. This procedure enabled the coders to identify any mismatches between the theme and subtheme names and the original data. Furthermore, the coders identified any themes, subthemes, codes, or data within codes that were deemed of low relevance to the overall themes or this study’s intention. This relevance-based scrutiny was followed by analysis of any codes that were characterized by very few participant quotes. This way, we were able to determine the *weightiness* or key ideas across the entire data set. In cases where relevance and the number of participant responses were considered to be low, the subtheme or code was discarded from the overall thematic map.

In the final phase of coding, the original coder and consulting coders collaboratively identified the main focus and extent of each theme. Some names of the main themes and subthemes were also modified to increase the alignment between the documented theme and subtheme labels and the data they each contained. This refinement of the theme and subtheme definitions informed the final write-up phase.

The Standards for Reporting Qualitative Research checklist was used as a guide for reporting (Multimedia Appendix 3) [21], and ethics approval was granted by the Avondale Human Research Ethics Committee (approval 2018.09).

## Results

### Overview

Most participants were female (262/320, 81.9%), and 50.3% (161/320) of the participants were aged 40 to 60 years. Almost one-third of the cohort (91/320, 28.4%) were aged 25 to 39 years, 15.6% (50/320) were aged 61 to 81 years, and 5.6% (18/320) were aged 18-24 years. Two-thirds of the cohort (212/320, 66.2%) worked either full or part time, 6.2% (20/320) identified as students, and the remaining 27.6% (88/320) identified as being unemployed or doing *home duties*. The majority of the cohort (240/320, 75%) provided a codable response describing facilitators of adherence, and almost all participants (314/320, 98.1%) provided codable responses related to barriers.

As the participants were asked to respond to 2 separate statements (one focusing on facilitators and the other focusing on barriers), responses to these 2 statements were analyzed separately. However, in some cases, participant responses overlapped and issues were reported as both a facilitator and a barrier. Broad themes that emerged were time, program components, personal factors, system design, technology, and human support. Notably, comment frequency regarding barriers to adherence were more than double the comments specifying facilitators of adherence. Table 1 (facilitators of adherence) and Table 2 (barriers to adherence) outline the thematic framework of responses.

During the development of the thematic map, it became evident that there was strong alignment between themes and subthemes in both sets of responses, that is, the thematic map illustrates the two-fold nature of most themes, with the order of reporting of the themes reflecting the most important themes first, as assessed by the coding researcher, according to relevance and volume of comments received. The issue of time emerged as a dominant theme in the data, with the vast majority of participants mentioning *time* across their responses.

**Table 1:** Perceived facilitators of adherence—themes, subthemes, and codes.

Themes, subthemes, and codes or category				Comments, n
**Time**				
	**Availability**			
		Opportune circumstances	20	
		Weekend	12	
	**Efficiency**			
		Priority and planning	14	
		Multitasking	10	
**Program components**				
	**Videos**			
		Highly engaging	70	
		Length	5	
	**Challenge or experiential factors**			
		Achievable	18	
		Enjoyable	12	
		Part of normal lifestyle	6	
		Accountability	5	
	**Overall program**			
		Easy or engaging	8	
		Quality	5	
**Personal factors**				
	**Motivation**			
		High interest in personal health	9	
		Commitment to complete	5	
	**Perceived value**			
		Personally beneficial	11	
		Reinforcement of positive habits	5	
**System design**				
	**Convenience**			
		Accessibility	16	
		App	14	
	**Usability**			
		Extra features	7	
		Ease of use	7	

**Table 2:** Perceived barriers to adherence—themes, subthemes, and codes.

Themes, subthemes, and codes or category				Comments, n
**Time**				
	**Daily life**			
		Lack of time in general	129	
		Work and study combination	58	
		Family responsibilities	40	
		Travel	16	
		Full schedule	8	
		Work and family combination	6	
	**Life event**			
		Death	9	
		Medical	6	
		Relocation	5	
		Other major change	5	
	**Period**			
		Christmas	15	
		Holiday travel	6	
**Program components**				
	**Challenge or experiential factors**			
		Perceived as difficult	33	
		Time consuming	22	
		Too many activities	19	
		Disliked recording or logging activity	18	
		Forgot to implement or record	10	
		Confusion	5	
	**Video factors**			
		Length	17	
		Unappealing content or style	10	
		Extra segments	7	
**Personal factors**				
	**Well-being**			
		Illness	16	
		Fatigue	10	
		Mental health distress or disorder	7	
		Stress	5	
	**Capacity**			
		Lack of motivation	18	
		Lagging behind	17	
		Not a priority, so forgot	16	
**System design**				
	Lock restrictions		10	
	Features missing		9	
	Gamification		8	
	Login and navigation		8	
	Video playback		5	
	Recording activity		5	
**Technology**				
	**Internet**			
		Internet and data access	28	
	**Personal device**			
		Faulty	18	
		Interrupted viewing	6	
**Human support**				
	Videoconferencing difficulties^a^		11	
	Lack of prompts		8	
	Lack of accountability		6	
	Preference for face-to-face		5	

^a^Videoconferencing support was provided to 1 subgroup during the intervention (n=103).

### Facilitators of Adherence

#### Time

Time was perceived as a facilitator of adherence when participants believed that they had time available and efficiency was optimized. Each weekly lesson was released on a Sunday, and this timing was viewed positively. Expediency afforded by being retired, living alone, or not working also facilitated adherence:

I liked that the videos were released on Sunday, even if some Sundays were busy and we couldn't make time to watch them that day. Sunday is a day it’s easier to make time to watch the full set of lesson videos.

ID 2614

Some participants prioritized, scheduled, and multitasked to ensure that the program components were completed each week. Specific factors that permitted time availability or efficiency included being at home, not traveling, completion of usual duties, incorporation of tasks into daily activities, a high level of organization or planning, and having children in a regular sleep routine:

When I made specific time to sit down and watch the program it made it easier to participate and plan how I can incorporate the challenges.

ID 2278

#### Program Components

This theme refers specifically to video content and the completion of practical activities. A total of 3 subthemes related to the program’s components emerged from the analysis: the overall program, video presentations, and experiential activity.

Although some participants perceived that the overall program was of high quality, easy to understand, and engaging, the key program components perceived as facilitators of adherence were the video presentations and the experiential challenges. Engaging video content has been frequently reported as a facilitator of adherence. The terms used to describe the videos included *humorous*, *entertaining*, *interesting*, *motivating*, and *easy to understand*. Participants appreciated the lighthearted presentation style, which included just enough information and interesting facts, to avoid information overload:

The videos were funny and engaging. Had enough info to be interesting but not overly filled with over-your-head stuff.

ID 2380

The daily and weekly experiential challenges that participants completed and recorded were perceived as fostering adherence because they were relevant, easy to accomplish, enjoyable, simple to log or record, and readily incorporated into daily life. Some challenges could be easily doubled up and, for some participants, recording their challenge activity provided a sense of accomplishment:

The small daily challenges were able to be absorbed into the day without having to find extra time and it felt like many could become habits.

ID 2302

I found a lot of the challenges crossed into another which made it easier such as being in the green and blue and going for a walk covered two challenges.

ID 2550

#### Personal Factors

There are numerous personal reasons that may influence adherence, including beliefs, motivation, personal well-being, and a broad array of diverse circumstances. During coding, several key personal subthemes were developed that included motivational factors, beliefs about the value or relevance of the program, level of personal well-being, and individual capacity.

Motivation and perceived value were identified as subthemes that positively influenced adherence at a personal level. Participants reported positive motivation through a high level of readiness, commitment to the intervention, and a personal desire to improve their health. Some participants were motivated to maintain their adherence to the program based on their belief that the intervention would be personally beneficial to them. They recognized a number of enabling aspects of the program, including the way it reinforced positive lifestyle choices; recognized that even small changes would be worthwhile; and identified the value of immediate effects. Participants also benefited from the multifactorial approach:

I looked forward to the video presentations because they were so relevant to my life, very interesting and well presented. I have been experiencing problems with sleep issues for many years and even taking herbal sleeping supplements, but after applying the information I received from the Live More sessions, I’ve been a good 7 to 8 hours sleep every night. This has been mainly the result of all the walking I’ve been doing - between 1 to 2 hours a day and more. I’m now taking more notice of nature and enjoying more green and blue. Having recently moved from [name of Country], I had no friends and felt lonely. So I went to the local Library and Community Centre and got a booklet with the different activities for retired people. I joined a group of like-minded people and enjoyed the activities they provided.

ID 2419

#### System Design

This theme relates to the various design and accessibility features of the web or mobile app and primarily includes the methods used to disseminate weekly video content and record experiential activities in the digital setting. Additional features such as gamification (ie, earning points), the ability to view what others were doing, and accessibility to extra resources were also included. Similar to other themes, the system design facilitated adherence for some participants and was perceived as a barrier for an almost equal number.

Two key design concepts were reported by users as positive influences on adherence: accessibility and convenience of the mobile app. Participants appreciated flexible access to view and review the content. This was amplified with easy portability through a mobile app:

I could do it when it was suitable for me. Whether 10pm or 6am. The flexibility made it possible.

ID 2195

### Barriers to Adherence

#### Time

Participants overwhelmingly perceived lack of time as problematic to adherence, with noticeably more comments referencing time as a barrier to adherence rather than a facilitator. Furthermore, time was mentioned substantially more often than any other factor recognized as a barrier. The 3 subthemes generated in relation to lack of time were daily life, life events, and the intervention period.

The demands of daily life were the foremost barriers and predominantly included work, study, and family commitments and the various combinations of all three:

Small child needing attention, lack of help from spouse due to work commitments; other extra-curricular activity; helping my elderly parents with household tasks and other daily activities.

ID 2538

Major life events were perceived as hindering adherence and included the death of a loved one, relocation, major illness, or a combination of trials that consumed major amounts of time:

The timing of this project was the one of the busiest 10 weeks in my life! Moved house. Solo parented for 4 weeks. My sister’s wedding. School holidays. The sickest our family has been in 10 years. Term 4 after-hours school events as part of my work responsibilities. We moved house the week the program started and then went straight into school holidays of which we were away the whole time. Then we were really sick for three weeks whilst I was solo parenting. So I just never got the chance to get started.

ID 2214

The intervention was conducted from September to early December 2018. The lead up to Christmas and the added pressures that often accompany the Christmas period in the work and social environment were deemed barriers. In addition, for this cohort (ie, a faith-based population), church activities were perceived as adding further to the time pressures already experienced at this time of year:

Making a commitment at this time of year was more difficult than I thought; this time of year is hectic for my job which also probably means my answers at the end could actually be worse than at the start, because of how tired I have been.

ID 2630

If it was at another time of year I probably would have completed it. This time of year is crazy for me at work, I'm a preschool assistant, and I am involved with helping with Road to Bethlehem [Christmas program]. The last few weeks I just found it difficult to think much about what I was doing.

ID 2417

#### Program Components

Some users deemed the overall program too intense and too long. Video segments were perceived as lengthy, and some users found the content unappealing and disliked the presentation style, describing it as *cliché*, *corny*, and *over the top*:

The videos were too long. I'd prefer 2-3 shorter ones per topic. That would be easier to watch while in train or fitting between other things I had to do.

ID 2318

While the actual science was interesting, much of the video content felt patronising and the jokes fell flat.

ID 2142

Most perceptions about the program components were directly related to difficulties with experiential activities. Time was viewed as a hindrance for both completing and logging the activity, and some participants perceived the activities as being too hard. Although some participants completed the daily and weekly experiential challenges, they disliked recording their activities, felt overwhelmed by the increasingly growing list of activities to record, or simply forgot to log their activities:

Having the challenges compound each week to record them meant that it was too overwhelming and I gave up logging on to record. I think having reminders about the previous weeks challenges is great, but filling in that many challenges every day is too hard!

ID 2287

#### Personal Factors

Numerous personal factors hindering adherence were grouped under 2 subthemes: personal well-being and capacity. Personal illness was the dominant factor affecting well-being. Other factors influencing well-being included the presence of a mental disorder, stress, and fatigue, with several respondents specifically identifying screen fatigue. Comments regarding capacity were equally divided among 3 key areas: a reported lack of motivation, discouragement at lagging behind in viewing content or completing experiential activities, and simply not prioritizing the intervention:

When I had downtime I didn't have the motivation to intently watch the videos & do the thinking & work required so it was easy to fall behind.

ID 2204

Also, with working full time (looking at a screen) all day, I really didn't want to sit in my home office to look at another screen, so I failed to keep on top of the weekly presentations.

ID 2563

#### System Design

Several design features and difficulty navigating the website were perceived as negative influences on adherence. Most comments were made in relation to the lock restrictions, the lack of some desirable features, dissatisfaction with the gamification setup, and the perceived tedious system for logging experiential activity. Although participants were provided with a recommended 10-week schedule for engaging with the intervention content, some participants emphatically opposed the lack of autonomy in how and when they consumed the information:

I don’t mind having to unlock videos - just please don’t constrict it to a week at a time!!! And let me do any or all of the challenges at the start or when I unlock things in my own time - don’t make me wait 7 days / a fortnight / 3 weeks etc to engage in all the challenges just because! Let me unlock them at my pace.

ID 2142

Desirable features that were noted as missing included an audio-only option, the ability to predownload the video content, and the option to read the material instead of viewing a video. A readable script was actually provided, although it was not explicitly recommended as an alternative to viewing video content. Although gamification was included as a way to enhance adherence, for some participants it was perceived negatively and a deterrent to further adherence:

It's very demotivating having a leaderboard for someone who's not near the top.

ID 2549

The system setup for recording experiential activity was seen as a barrier because it was perceived as difficult to use, with one participant describing it as “glitchy and not particularly intuitive” with “long and clunky lists*”* [ID 2142]:

Prompts on challenge input boxes were too vague about what the user needs to write. Should the user explain anything or just state that the challenge was completed? It was not clear whether the user's statement in the challenge input box would we shared with others (user had to just try it and see what happens during the first session). I could not log more than one challenge on the interface at a time (I had to change to another page and then come back to the challenges to log another one).

ID 2391

#### Technology

Technology (ie, data availability, internet access, and device suitability) was deemed a barrier, mainly because of poor internet accessibility or personal device problems.

Internet access problems included slow download speeds, internet outages, and no internet access at home. Personal device problems included old or faulty smartphones that could not download the mobile app, small screen size, sound problems, and perceived inability to correctly operate the device:

We had some difficulty watching the videos on my tablet, the volume is not loud enough, we really need a PC or to data project to a bigger screen for optimal viewing/listening.

ID 1767

#### Human Support

Human support offered to participants varied depending on the group allocation they received. All participants received automated email reminders to engage with video content and record their practical activities. One group received regular text messages, and the other group was offered weekly videoconference sessions as an extra measure of support. Despite human support being the focus of the original study [16], perceptions about the support offered did not feature strongly. However, some participants reported that adherence was impeded because of human support factors or lack thereof. A lack of prompting and accountability were stated as reasons for difficulty in adherence. In the group that received videoconferencing support, some of the group members reported that videoconferencing support was problematic for a range of reasons, most commonly that the meetings were held at an unsuitable time:

Timing of online groups sessions didn’t work for me with my responsibilities during that time period. I needed a later evening session. 9pm! Or a 5:30am session. The timing of the first 5 weeks would have been difficult for me regardless.

ID 2214

## Discussion

### Principal Findings

This study provides novel insights into participants’ perspectives regarding facilitators and barriers to adherence in a digital MHPI for a community-based nonclinical population. Themes often had dual impacts, deemed as both facilitators and barriers. Facilitators of adherence included engaging in video content, achievable experiential activities, time availability, user-friendly web and mobile app design, and high personal interest or motivation. Conversely, a perceived lack of time was the main barrier to adherence. Completing and logging practical activities, a range of personal influences, problematic design features, technological issues, and a lack of support or prompting were also identified as barriers.

As barriers were the predominant focus of participants’ perspectives, it is clear that an MHPI is a difficult endeavor for a nonclinical group and that time is the foremost issue in relation to adherence. Similarly, other qualitative studies in workplace settings [14,15] have concluded that time scarcity is a major inhibitor of adherence and that participants need interventions requiring only small time commitments. It is evident that certain features or problems (eg, difficult website navigation and restrictions on video viewing) consumed inordinate amounts of the participants’ time, thereby negatively impacting their adherence. Similarly, such issues have previously been exposed as barriers to the success of web-based learners in educational settings [22,23]. Therefore, time efficiency must be a core consideration by researchers in every aspect of the intervention design. It is vital to design interventions to minimize time and energy investment for participants while concurrently optimizing outcomes.

An important element that underpinned the data was the participants’ desire to easily incorporate core components of the program (ie, videos and challenge activities) into their everyday lives. A key priority in the design and development of future interventions should be to collaborate with potential users in designing interventions that enable autonomous, optimal integration of the essential intervention elements into daily life. Practical examples may include the provision of multiple options to access the content (eg, video, print, or audio), choice of methods to log activities, and recommendations on combining some activities to make optimal use of time.

The preponderance of dual themes in this study sheds more light on an important element that has been identified previously [24], that is, individual differences have a substantial influence on adherence to MHPIs. Although previous research by Banerjee et al [13] identified several twofold themes in a qualitative study focusing on engagement with a mindfulness intervention, a study by Zarski et al [24] on adherence to a digital stress management intervention noted that, even after accounting for socioeconomic demographics, personal well-being status, and human support, many individual variances in nonadherence were inexplicable. This study highlights some of these differences.

Understandably, the high prevalence of individual variance confounds efforts to crystallize a precise set of factors influencing adherence to digital interventions. Plausibly, future research should embrace individual differences by examining ways to prioritize participant autonomy in tailoring interventions according to personal preferences. This could be achieved by offering an eclectic, self-directed approach that encourages participant choice in activating or deactivating a range of optional features (eg, gamification or type and frequency of human support) as desired. This constructivist-style approach, commonly used in educational settings, is well supported by research as an efficacious method [25,26].

The original study, from which this qualitative study was derived, focused on the influence of varying levels of human support on outcomes and adherence to an MHPI [16,17]. However, in this qualitative analysis, human support did not have a strong influence on adherence. Considering that the quantitative analysis revealed that adherence was not impacted by different levels of support and that participants achieved improvements in outcomes irrespective of the human support provided, this is not surprising. Although numerous studies have demonstrated that human support may improve outcomes [9,27,28], there is substantial evidence that well-designed, self-guided interventions can be successful without added human support [9,29-32] and their associated costs.

### Strengths and Limitations

The large cohort and inclusive age range (18-81 years) of participants were strengths of this study. Furthermore, participants were not restricted to the length of their responses and could write as little or as much as desired. All comments were coded, and participants received no guidance on what topics to comment on, eliminating preconceptions by researchers.

Recruitment bias could be seen as a potential limitation to this study as participants were self-selected into the study, and the target group were Seventh-day Adventist Church members who typically share common health practices (eg, abstinence from alcohol or smoking) and may be more inclined to take a greater interest in health than the general population. In addition, recruits were largely tertiary educated (262/320, 81.9%), White (280/320, 87.5%), and female (262/320, 81.9%). Although a high female proportion is the characteristic of an internet intervention [33,34], it limits transferability to the wider population. In addition, participants were not given the opportunity to indicate whether they used the web-based platform, the mobile app, or a combination of both, which prohibited a comparison of data based on the different delivery methods.

By allowing participants to comment freely on an open-ended statement, the assumption must be made that participants only reported on factors most relevant to their personal experiences. Therefore, the study does not contain all the views of the participants. As researchers, we adopt the position that the predominant issues are exposed and that the volume of comments depicts saturation on crucial issues.

Notably, 2 other factors may have inadvertently influenced participants’ perceptions. First, barriers, particularly *time*, may have been augmented by the period that the intervention was conducted (mid-September to early December 2018). Avoiding administering an intervention that ends in December is a learning for future implementation. Second, the website and mobile app design, in permitting recording of an increasing array of experiential activities as the weeks progressed (rather than just the activities for the current week), may have augmented dissatisfaction with the logging or recording component, introducing an element that went beyond the intentions of the original intervention design.

### Conclusions

Although there are a diversity of factors influencing adherence, highly engaging content encourages adherence and time efficiency is a principal consideration for a nonclinical, community-based cohort. Therefore, intervention designers should place a concerted focus on streamlining interventions to reduce the time required by participants and simplify all program elements. Other considerations to increase adherence include allowing participants to create their own experience by choosing what optional features to include or exclude, enhancement of the website and mobile app to facilitate a more intuitive experience, shorter video presentations that are easily accessible and provide high autonomy in the way they are consumed, simplified and limited recording of experiential activities, and options for personal choice in accountability. In future research, it will be important to consider the views of users in relation to adherence and respond with an adequate level of agility that assures an ever-improving experience for participants to ensure optimal adherence and sustainability.

## Appendix

Multimedia Appendix 1 Program overview.

Multimedia Appendix 2 Web or app screenshots.

Multimedia Appendix 3 Checklist—standards for reporting qualitative research.

## Abbreviations

eLMS: web-based learning management system
MHPI: mental health promotion intervention
RTA: reflexive thematic analysis
