# Supplementary material for: Participant Perceptions of Facilitators and Barriers to Adherence in a Digital Mental Health Intervention for a Nonclinical Cohort: Content Analysis
Source: J Med Internet Res. 2021 Apr 14;23(4):e25358. doi: 10.2196/25358 (PMC8082377; doi:10.2196/25358)
Supplement: Multimedia Appendix 1 [file jmir_v23i4e25358_app1.pdf]

Live More Project – Program and experiential activity overview

Week	Topic	Overview	Daily experiential activity	Weekly experiential activity
1	Speak positively.	Introduction to limbic system, positive language.	Offer a genuine compliment.	Memorise an inspirational text/saying.
2	Move dynamically.	Physical activity.	30 minutes of moderate-intensity activity.	Perform resistance exercises (video provided).
3	Immerse in an uplifting natural environment.	Natural landscapes, light.	Spend 30 minutes outside in natural light.	Watch a sunrise from an appealing location.
4	Immerse in a positive social environment.	Positive relationships, forgiveness.	Perform an act of love for a person, using their love language.	Act of forgiveness or establish a new friendship.
5	Look to the positive.	Focus on positive, upward and downwards spirals.	Write down 3 things that went well.	Gratitude visit: write a letter, deliver and read to the person.
6	Eat nutritiously.	Gut health, plant-based foods, fibre.	Eat eight fists full of fibre.	Prepare and share a plant-based meal with one or more friends.
7	Rest – sleep.	Sleep: 7-8 hours, blue light vs. yellow/orange light, factors affecting sleep.	Spend 8 hours in bed.	Spend an evening by fire or candlelight.
8	Rest – from stress.	Strategies to de-stress, rest day, circaseptan rhythms.	'Sit in silence' mindful activity (15 mins).	Take a 'guilt-free' day off.
9	Serve others.	Serving - emotionally uplifting, serve sustainably using signature strengths.	Perform one or more random acts of kindness.	Use significant strength to perform a significant act of service.
10	What does it take to flourish?	Five areas to flourish: PEARM – Positive emotions, engagement, achievement, relationships, meaning.	Take time to engage in something you enjoy.	Create a list of goals and an action plan to achieve them.
